# A Simplified Positive-Sense-RNA Virus Construction Approach That Enhances Analysis Throughput

**DOI:** 10.1128/JVI.02261-13

**Published:** 2013-12

**Authors:** Bunpote Siridechadilok, Methee Gomutsukhavadee, Thunyarat Sawaengpol, Sutha Sangiambut, Chunya Puttikhunt, Kwanrutai Chin-inmanu, Prapat Suriyaphol, Prida Malasit, Gavin Screaton, Juthathip Mongkolsapaya

**Affiliations:** National Center For Genetic Engineering and Biotechnology (BIOTEC), National Science and Technology Development Agency, Pathumthani, Thailanda; Bioinformatics and Data Management for Research Unit, Office of Research and Development, Faculty of Medicine, Siriraj Hospital, Mahidol University, Bangkok, Thailandb; Dengue Hemorrhagic Fever Unit, Office of Research and Development, Faculty of Medicine, Siriraj Hospital, Mahidol University, Bangkok, Thailandc; Molecular Immunology Unit, Hammersmith Campus, Department of Medicine, Imperial College London, London, United Kingdomd

## Abstract

Here we present an approach that advances the throughput of a genetic analysis of a positive-sense RNA virus by simplifying virus construction. It enabled comprehensive dissection of a complex, multigene phenotype through rapid derivation of a large number of chimeric viruses and construction of a mutant library directly from a virus pool. The versatility of the approach described here expands the applicability of diverse genetic approaches to study these viruses.

## INTRODUCTION

An important genetic tool to study animal, positive-sense RNA viruses is the infectious clone, a form of viral genome cloned on a plasmid that can be propagated in Escherichia coli and manipulated for reverse-genetics analysis. While the tool has yielded important insights into viral infection and is critical for generating vaccine candidates, the instability of certain viral genome sequences in E. coli (especially in the cases of most flaviviruses [1] and coronaviruses [2] and of some picornaviruses [3], togaviruses [4], and pestiviruses [5]) has limited the scale of genetic analyses for these viruses. The instability is still not well understood. Consequently, each mutant construct must be extensively sequenced to verify the absence of adventitious mutations, potentially resulting in months of efforts to establish one such clone. Though there are many cloning approaches to mitigate the instability (6–11), a large-scale genetic analysis relying on cloning methods still requires significant efforts to establish the mutant or chimeric viruses. In addition, the cloning methods are not adept at capturing the diversity of a virus pool for manipulation and screening, hindering the application of available genetic approaches to study viruses.

**Fig 1:**
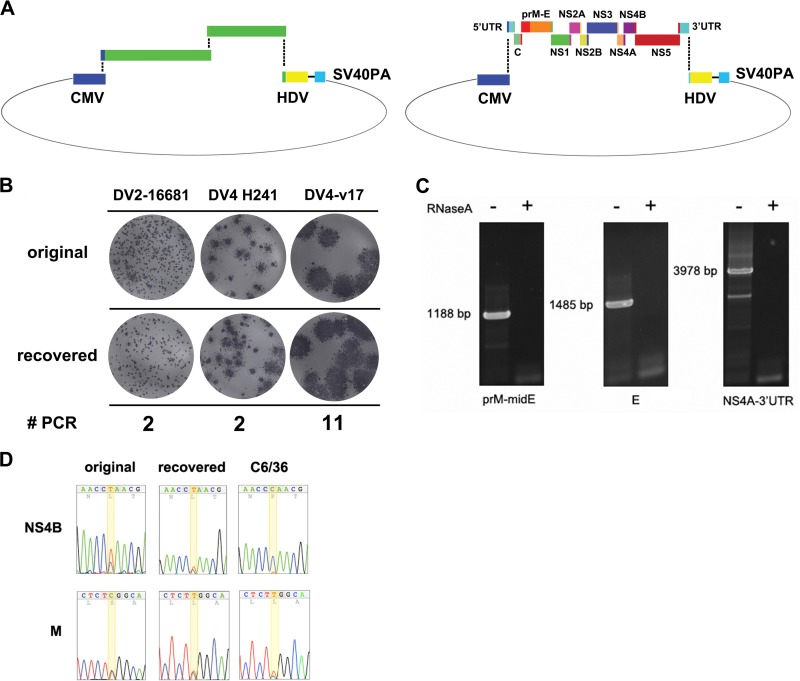
Construction of DENV by Gibson assembly. (A) Assembly schemes of DENV infectious DNA. CMV, CMV promoter; HDV, HDV ribozyme; SV40PA, polyadenylation signal. (Left) Assembly scheme for DENV2-16681 and DENV4-H241; (right) assembly scheme for DENV4-v17. (B) The comparison between the foci of the original virus stocks and the foci of viruses recovered through Gibson assembly. The number of DENV PCR products assembled (# PCR) is shown. (C) PCR products for sequencing of virus recovered from transfected 293T cells were generated from cDNA and not contaminated assembled DNA. +, viral RNA was treated with RNase A during cDNA synthesis. (D) Comparison between sequence heterogeneities of the original v17 strain, v17 recovered by our method, and v17 cultured in C6/36 cells. The heterogeneous positions are highlighted in yellow.

Here, we describe a simple virus construction approach which could bypass cloning and which eliminated its limitations in a large-scale genetic analysis of dengue viruses (DENV). The DENV genome could be reconstructed from multiple PCR products (amplified from cDNA) in a single DNA assembly reaction. Unlike the conventional DNA ligation method, DNA assembly by Gibson assembly (12), which was used in this technique, stitches DNA through overlapping, homologous sequences at the ends of DNA fragments, thus negating any need to introduce foreign sequences to accommodate ligation and providing the flexibility of being able to join DNA at any locations on the viral genome. To produce virus from DNA, viral PCR products, amplified from viral cDNA using high-fidelity polymerase with proofreading activity, were assembled onto an expression plasmid with a cytomegalovirus (CMV) promoter that precisely initiated transcription on the virus genome sequence and a terminator, such as hepatitis D virus (HDV) ribozyme, that accurately generated 3′ end of the transcribed viral RNA (13, 14) (Fig. 1A).

## MATERIALS AND METHODS

### Cells and virus.

293T cells were maintained in Dulbecco's modified Eagle medium (DMEM) supplemented with 10% heat-inactivated fetal calf serum, 1 mM glutamine, 1 mM sodium pyruvate, 20 mM HEPES, and high glucose (4.5 g/liter). C6/36 cells were maintained at 28°C in L15 supplemented with 10% tryptose phosphate broth, 1 mM glutamine, and 10% heat-inactivated fetal calf serum. Vero cells were maintained at 37°C with 5% CO_2_ and 80% humidity in MEM supplemented with 1 mM glutamine and 10% fetal-calf serum. DENV4-H241 and DENV2-16681 were cultured in C6/36 cells that were maintained in L15 supplemented with 10% tryptose phosphate broth, 1 mM glutamine, and 1.5% heat-inactivated fetal calf serum after infection. All media used in this study were also supplemented with penicillin/streptomycin.

DV4 strain v17 was generated by serial passaging of DENV4-H241 in Vero cells for 17 passages. DV4 strain 4.1 was isolated in the form of an infectious clone constructed from DENV4-H241 virus stock. The construct of DENV4 strain 4.1 was cloned and propagated in E. coli XL10 Gold strain (Agilent) cultured at 22°C in LB medium.

### Construction of the expression plasmid.

A CMV promoter was obtained by PCR amplification from pcDNA 3.1(+) Hygro (Invitrogen). HDV ribozyme and SV40 PA (sequence based on the work by Varnavski et al. [14]) was synthesized and cloned on a high-copy-number vector with flanking NheI and BamHI sites (Invitrogen). The CMV promoter, HDV ribozyme, and SV40PA were assembled onto a pUC19 backbone first with In-fusion HD (Clontech) and later by conventional restriction ligation (NheI and BamHI sites). The plasmid construct was verified by sequencing.

### cDNA synthesis and PCR amplification.

Viral RNA was extracted from culture media with QIAamp viral RNA extraction kit according to the manufacturer's protocol (Qiagen). cDNA synthesis of the virus was carried out with Superscript III first-strand synthesis kit (Invitrogen) according to Chin-inmanu et al. (15). The primers for cDNA synthesis were 10601-10621-rv-dv4 and 10639-10661-rv-dv2 for DENV4 and DENV2, respectively. Primer sequences are shown in Table 1.

**Table 1:** Primers used in construction of viruses

Primer name	Sequence (5′ to 3′)
CMV-5′ UTR-DV4	AGAGCTCGTTTAGTGAACCGAGTTGTTAGTCTGTGTGGACCGACAAGGAC
CMV-5′ UTR-DV2	AGAGCTCGTTTAGTGAACCGAGTTGTTAGTCTACGTGGACCGACAAAGACAG
DV4-6671-6698-rv	ATGATTGAGGCCGCTATCCACTG
DV4-6671-6698-fw	TGGATAGCGGCCTCAATCATACTAGAG
DV2-6685-6709-rv	CCAGTATTATTGAAGCTGCTATCCA
DV2-6685-6709-fw	CTTCAATAATACTGGAGTTTTTTCTCATAG
3′ UTR-DV4	AGAACCTGTTGGATCAACAACACCAATCCATCTCGCGGCGTTCTGTGCCTGGAATGAT
3′ UTR-DV2	AGAACCTGTTGATTCAACAGCACCATTCCATTTTCTGGCGTTCTGTGCCTGGAATGAT
hCMV-rv	CGGTTCACTAAACGAGCTCTGCTTATATAGACCTCCCACCG
HDV-fw-dv4	TTGTTGATCCAACAGGTTCTGGGTCGGCATGGCATCTCC
HDV-fw-dv2	CTGTTGAATCAACAGGTTCTGGGTCGGCATGGCATCTCC
10601-10621-rv-dv4	CGGCGCTCTGTGCCTGGATTG
10639-10661-rv-dv2	TGGCGTTCTGTGCCTGGAATGAT
C-dv4-start-rv	CTAACCACCTTTTTTCGTTGGTTCAT
C-dv4-start-fw	ATGAACCAACGAAAAAAGGTGGTTAG
prM-dv4-start-rv	CGCCATCTCTTGTTGACAAGTG
prM-dv4-start-fw	CACTTGTCAACAAGAGATGGCG
E-dv4-start-rv	TCCCCACTCCCACGCATCG
E-dv4-start-fw	CGATGCGTGGGAGTGGGGA
E-dv4-end-rv	GTCTGCGTGAACTGTGAAACCC
E-dv4-end-fw	GGGTTTCACAGTTCACGCAGAC
NS1-dv4-end-rv	GGCCGATACCTGTGATTTGACC
NS1-dv4-end-fw	GGTCAAATCACAGGTATCGGCC
NS2A-dv4-end-rv	TCTCTTTGAAGCTCCTTTCATGAGAGTC
NS2A-dv4-end-fw	GACTCTCATGAAAGGAGCTTCAAAGAGA
NS3-dv4-start-rv	GACGTCCCACAGGGCTCCTGA
NS3-dv4-start-fw	TCAGGAGCCCTGTGGGACGTC
NS3-dv4-end-rv	CTTTCTTCCACTGGCAAACTCCTTG
NS3-dv4-end-fw	CAAGGAGTTTGCCAGTGGAAGAAAG
NS4A-2K-dv4-end-rv	GGCTGCTATGAGACCAATAATGGTG
NS4A-2K-dv4-end-fw	CACCATTATTGGTCTCATAGCAGCC
NS5-dv4-start-rv	GTCTCTCCTGTGGTCCCAGTTCC
NS5-dv4-start-fw	GGAACTGGGACCACAGGAGAGAC
NS5-dv4-end-rv	CAGAACTCCTTCACTCTCGAAAGG
NS5-dv4-end-fw	CCTTTCGAGAGTGAAGGAGTTCTG
midE-dv4-1587-1610-rv	CCAATGAACTTCTGATGTGTCTGC
midE-dv4-1587-1610-fw	GCAGACACATCAGAAGTTCATTGG
M-dv4-start-rv	CATTCCTGAATGTGGTGTTAGGGCTA
M-dv4-start-fw	TAGCCCTAACACCACATTCAGGAATG
Post-E1690-fw	GTGCTAGGATCTCAGGAAGGAGC
E1690-random-dv4-rv	GCTCCTTCCTGAGATCCTAGCACNNNCACATCCTGTCTCTTGGCATGAG

PCR products for assembly of viral constructs were carried out with high-fidelity DNA polymerases (Phusion [NEB] and KAPA HiFi [KAPA Bioscience]) according to the manufacturers' protocols. All the PCR products were cleaned up using a PCR cleanup kit from Invitrogen before use in DNA assembly and sequencing.

PCR products of the expression vector (for assembly with viral PCR products) were amplified with hCMV-rv and HDV-fw-dv4 (for DENV4) or HDV-fw-dv2 (for DENV2).

PCR amplifications of cDNAs of DENV4-H241 (and DENV2-16681) genomes were carried out by two pairs of primers: CMV-5′ UTR-DV4 (DV2) and DV4-6671-6698-rv (or DV2-6685-6709-rv) for the first half of the genome; DV4-6671-6698-fw (or DV2-6685-6709-fw) and 3′ UTR-DV4 (or DV2) for the second half of the genome. PCR was performed with Phusion polymerase (NEB).

Eleven PCR products were generated from v17 cDNA by 11 pairs of primers: CMV-5′ UTR-DV4 and C-dv4-start-rv (5′ untranslated region [UTR]), C-dv4-start-fw and prM-dv4-start-rv (C), prM-dv4-start-fw and E-dv4-end-rv (prM-E), E-dv4-end-fw and NS1-dv4-end-rv (NS1), NS1-dv4-end-fw and NS2A-dv4-end-rw (NS2A), NS2A-dv4-end-fw and NS3-dv4-start-rv (NS2B), NS3-dv4-start-fw and NS3-dv4-end-rv (NS3), NS3-dv4-end-fw and NS4A-2K-dv4-end-rv (NS4A), NS4A-2K-dv4-end-fw and NS5-dv4-start-rv (NS4B), NS5-dv4-start-fw and NS5-dv4-end-rv (NS5), and NS5-dv4-end-fw and 3′ UTR-DV4 (3′ UTR).

### Virus construction and production.

Cleaned-up PCR products of both the expression vector and viral cDNA were assembled together in a Gibson enzyme mix according to the one-step isothermal assembly protocol detailed by Gibson et al. (12). For this study, the overlaps between fragments to be assembled were in the range of 20 to 40 bp. The formula of the enzyme mix for overlap between 20 to 150 bp was used for the assembly (12). PCR products of the expression vector (0.02 to 0.04 pmol) were assembled with 0.04 to 0.08 pmol of each PCR product of the viral genome in the enzyme mix to make up 20 μl of the assembly reaction mixture. The Gibson ligation reaction mixture (7 to 20 μl; approximately 40 to 120 ng of mixed PCR products) was diluted in Opti-MEM I medium and mixed with Lipofectamine 2000 according to the protocol provided by manufacturer (Invitrogen). 293T cells in either 35-mm dishes or 24-well plates were washed twice with Opti-MEM before addition of the DNA-Lipofectamine complex solution to the cells. Transfection was performed for 4 h at 37°C before the medium was changed to DMEM supplemented with 10% heat-inactivated fetal calf serum, 1 mM glutamine, 1 mM sodium pyruvate, 20 mM HEPES, and high glucose (4.5 g/liter). The cultured medium was harvested and replenished on the second and third days after transfection. For mapping and library construction experiments (see Fig. 3 and 4), ISF-1 (Biochrom) was used instead of DMEM. Harvested media were clarified of debris and dead cells by centrifugation at 3,000 × *g* and 4°C for 5 min. The clarified medium was stored at −70°C for subsequent infection, virus titration, and viral RNA extraction for sequencing.

### Virus titration by focus-forming assay.

Vero cells (∼90% confluence) in 96-well plate were used for titration. Infection was carried out at 37°C for 3 h before the cells were overlaid with MEM supplemented with 2% fetal bovine serum (FBS) and 1.5% carboxymethyl cellulose. Infected cells were incubated at 37°C for 3 days before fixation with 3.7% formaldehyde in 1× phosphate-buffered saline (PBS) and permeabilized with 2% Triton X-100 in 1× PBS. Staining was performed with 4G2 as the primary antibody, horseradish peroxidase (HRP)-conjugated anti-mouse IgG as the secondary antibody, and DAB (3,3′-diaminobenzidine) as the chromogenic substrate.

### Focus quantitation.

Dried, stained virus titer plates were scanned and digitized with a KS ELISPOT reader (Carl Zeiss). Well images were extracted in Photoshop (Adobe). Foci in each well image were characterized in ImageJ (16). Well images were converted to binary form to separate foci and background. Then, the areas of foci in the binary image were quantitated (the number of pixels) using the “Analyze Particles” function.

### Sequencing of virus.

DNA sequencing of virus was performed with PCR products derived from viral cDNA. PCR amplification for sequencing was performed with either Phusion polymerase (NEB) or Accuprime high-fidelity *Taq* polymerase (Invitrogen). To control for the contamination of assembled DNA from transfection reaction, another cDNA synthesis reaction with RNase A (Fermentas) was also set up. PCR amplification of the cDNA plus RNase A did not yield specific PCR products, showing that PCR products were derived from RNA (Fig. 1C). The sequence chromatograms were analyzed and displayed using the 4Peaks program (Mekentosj).

### Growth curve comparison.

At least three independent experiments were performed for each virus tested for growth curve. The recovered viruses from Gibson assembly using DENV4-H241, DENV2-16681 and DENV4-v17 were compared against the original control viruses, and in the case of DENV2-16681, we also compared growth with that of virus produced from an infectious clone (a gift from N. Sittisombut). DENV2-16681 from the infectious clone, which had been derived from the transfection of capped *in vitro* transcribed viral RNA into C6/36 (17), was expanded in C6/36 before use. To perform growth curve comparisons, confluent Vero cells in 24-well plates were infected at a multiplicity of infection (MOI) of 0.25 (DENV2-16681 and DENV4-v17) or 0.01 (DENV4-H241) in 500 μl MEM supplemented with 2% heat-inactivated fetal calf serum. Infection was carried out for 2 h at 37°C with 5% CO_2_. After infection, the cells were washed three times with 1 ml plain MEM. The infected cells were then supplemented with 1 ml MEM supplemented with 2% heat-inactivated fetal calf serum. The medium from infected cells was collected for virus titration at every 24 to 26 h.

### Genetic mapping.

Eleven PCR products derived from strain 4.1 were generated by the set of primers used for strain v17. Another set of PCR products that cover two genes from both strains were also constructed to facilitate assembly using the same set of primers. Viruses in Fig. 3 were constructed from different combinations of 11 genes from both strains. To further map the mutations in E and prM, each gene was broken down with two pairs of primers. For E, the pairs E-dv4-start-fw plus midE-dv4-1587-1610-rv (E1527) and midE-dv4-1587-1610-fw plus E-dv4-end-rv (E1690) were used. For prM, prM-dv4-start-fw plus M-dv4-start-rv (prM550) and M-dv4-start-fw plus E-dv4-start-rv (M847) were used.

### Virus library construction.

To diversify the codon that encodes the amino acid specified at position E1690, E gene was reconstituted from two PCR products generated by two pairs of primers: E-dv4-start-fw plus E1690-random-dv4-rv and post-E1690-fw plus E-dv4-end-rv. These two PCR products were incorporated in the assembly reaction to construct diversified E gene for the viruses.

## RESULTS

### Recovery of dengue viruses with Gibson assembly.

**Fig 2:**
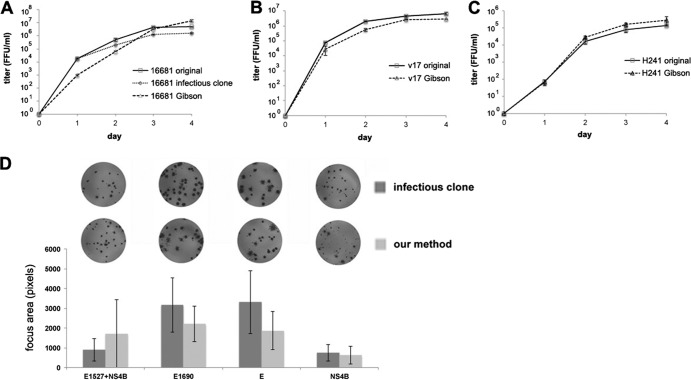
Growth comparison of DENV2-16681, v17, and H241 DENVs derived from Gibson assembly, conventional virus culture (original viruses), and the infectious clone (17). All the experiments were performed with Vero cells. (A-C) show the growth curves of DENV2-16681 (MOI = 0.25), v17 (MOI = 0.25), and H241 (MOI = 0.01), respectively. Error bar = standard error. (D) Focus comparison between the mutant viruses constructed from Gibson assembly of PCR products (bottom row) and by conventional infectious-clone method (top row). The viruses used in focus comparison correspond to the viruses with the same label in Fig. 3A (E and NS4B; left-hand panel), and 3D (E1690 and E1527+NS4B).

Transfection of 15 μl of the assembly reaction into 293T cells (seeded in 35-mm dishes at ∼250,000 cells/dish) using 10 μl of Lipofectamine 2000 and 500 μl Opti-MEM-I (Invitrogen) produced virus (up to 10^5^ focus-forming units [FFU]/ml) in culture media within a few days. Recovered virus had the same characteristic foci as the original virus stocks (Fig. 1B). The recovery depended on intactness of assembled DNA, as determined by excluding a genome segment or treatment with DNase before transfection inhibited virus production (data not shown). The viruses could be produced from as many as 11 viral PCR products (Fig. 1B, strain v17). DNA sequencing of the recovered DENV4-v17 showed sequence heterogeneities at two positions, as observed in the original DENV4-v17 stock used to derive its template cDNA, while further culture of DENV4-v17 in C6/36 cells, a mosquito cell line usually used for DENV isolation and recovery, lost the NS4B mutations (Fig. 1C). The heterogeneities in focus size and sequence of the recovered viruses demonstrated the ability of this technique to retain a pool of virus mutants. In addition to similar focus characteristics, the recovered viruses also possessed growth curves similar to those of either the original viruses used as PCR templates or virus derived from an infectious clone, in the case of DENV2-16681 (Fig. 2A to C).

In addition to recovery of a heterogeneous viral pool, Gibson assembly could be used for efficient construction of mutant viruses. To date, over 100 mutant DENVs (in addition to the ones presented here) have been constructed by this approach. Sequence verification by the Sanger method (10 viruses fully sequenced and the rest sequenced on the mutant genes and the assembled sites) confirmed that the desired mutations were obtained in all cases. A comparison between the foci of a set of mutant viruses generated from Gibson assembly and from a sequence-verified infectious clone also showed similar phenotypes (Fig. 2D).

### Convenient shuffling of viral genome segments for characterization of mutations.

The ability to conveniently and accurately construct a virus from a set of multiple PCR products or DNA fragments, with each representing a viral gene or genetic element (as shown for strain v17 in Fig. 1B), would greatly facilitate genetic mapping. It enables convenient derivation of numerous chimeric viruses from shuffling a set of PCR products or DNA fragments of genes/loci of two viruses. Instead of having to verify the DNA sequence of each full-length chimeric clone, as required by the infectious-clone approach, the shuffling requires sequencing of only two sets of viral DNA templates, greatly cutting down sequence verification in a large-scale mapping effort. In addition, the ease of recombining different virus strains offered by this scheme would provide a powerful basis that is not natural for single-genome RNA viruses but is indispensable for forward genetics of many organisms and viruses with segmented genomes.

To demonstrate the utility of this technique in mapping, we “crossed” two dengue strains with large (strain v17) and small (strain 4.1) foci by shuffling PCR products of their genes or loci to produce chimeric viruses. The viruses produced directly from transfected 293T cells were directly used for phenotype characterization to minimize the effects from random PCR errors that could be enriched or amplified during subsequent virus culture to expand the virus stocks. We characterized the foci of progeny viruses to determine which genetic differences between the two (Table 2) conferred their phenotypes. Focus size is an indicator of how well a virus replicates. A genetic variant that changes focus size can affect the replication of the virus.

**Table 2:**
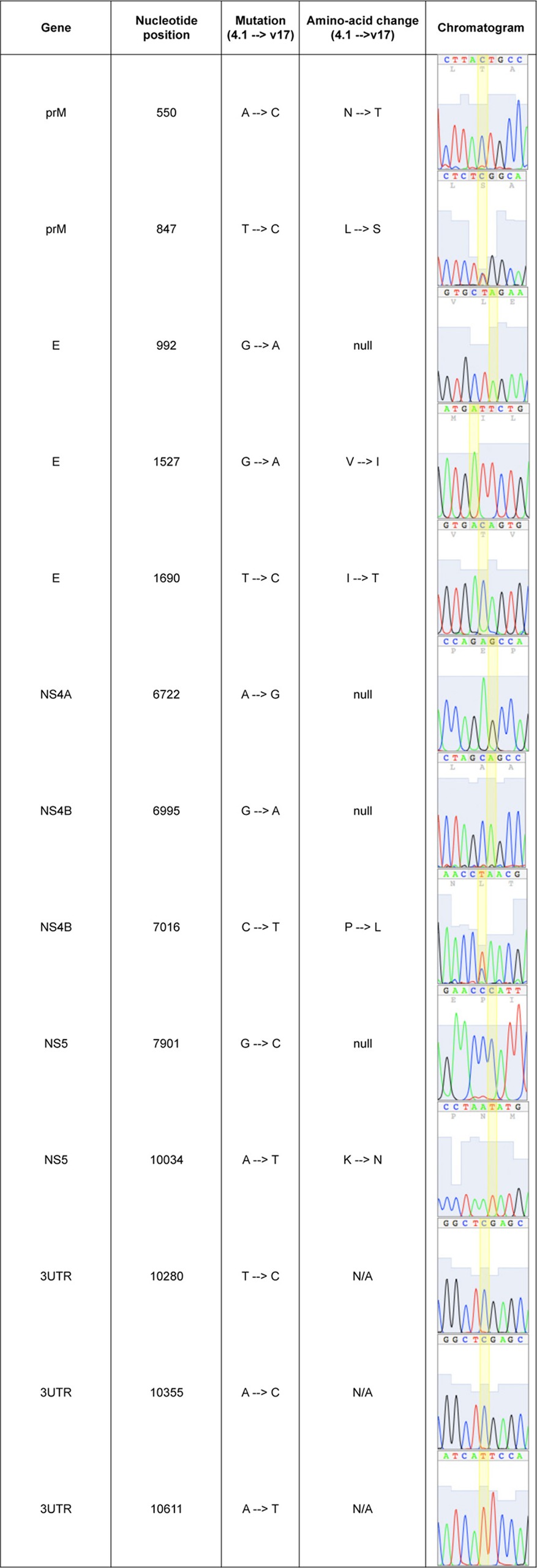
Summary of sequence differences between strains v17 and 4.1^*a*^

a Nucleotide differences are highlighted in yellow.

**Fig 3:**
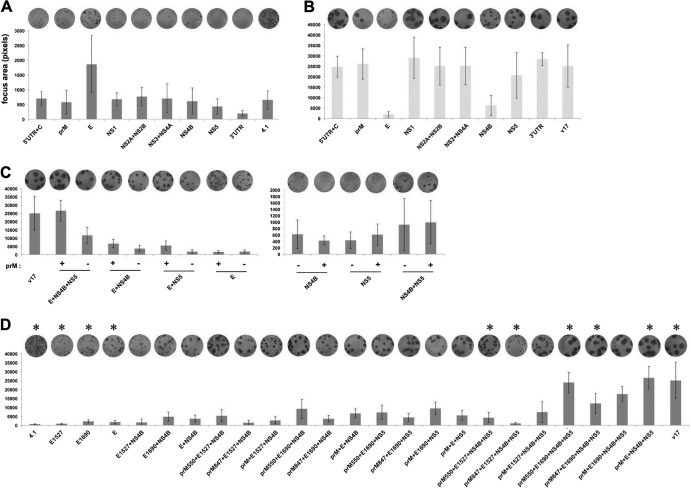
Crossing small-focus (strain 4.1) and large-focus (strain v17) viruses to map the mutations that confer large foci. (A) Foci of the chimeric viruses with a single segment or gene (*x* axis) of v17 replaced with the one from the other strain in the 4.1 background. (B) Reverse of the single-gene swaps (A). The v17 gene/segment was replaced with the corresponding one from 4.1. The focus sizes were measured as the average of the number of pixels occupied by the foci (*y* axis). Error bars show standard deviations. Well images from focus assays of the viruses are shown above the bars. (C) Foci of the chimeric viruses with the combinations of genes in strain 4.1 replaced with the ones from v17. (D) The influences of v17 mutations at positions prM550, prM847, E1527, and E1690 in reconstituting v17 focus size in the 4.1 background. The asterisks indicate the viruses that implicate the roles of the v17 mutations at prM550 and E1690 in retrieving v17 foci.

The mapping was done in three stages. First, we characterized the chimeric viruses derived from single-gene swaps to identify target genes (Fig. 3A and B). Replacement of either E or NS4B in v17 with the sequences from 4.1 caused a dramatic reduction in focus size (Fig. 3B). The reverse experiment, where single segments from v17 were moved into the 4.1 background, suggested a role for E which increased focus size but not to the level seen in v17 (Fig. 3A).

To characterize further elements contributing to the focus sizes of v17 and 4.1, we went on to make and characterize a panel of v17 gene combinations inserted into 4.1 that could retrieve the phenotype. We found that the combination of prM, E, NS4B, and NS5 of v17 could reconstitute the large v17 foci (Fig. 3C). Since there are two nonsynonymous differences between 4.1 and v17 in the E and prM sequences each (E at positions E1527 and E1690; prM at positions prM550 and prM847) (Table 2), we tested which of them could account for the effect of those two genes. We constructed additional 18 chimeric 4.1 viruses with single or combined changes at these positions. The focus sizes of a subset of these viruses (Fig. 3D, asterisks) suggested that the v17 mutations at position prM550 and E1690 could replace the prM and E of v17, respectively, in reconstituting v17 foci (Fig. 3D).

### Construction of a dengue virus library.

**Fig 4:**
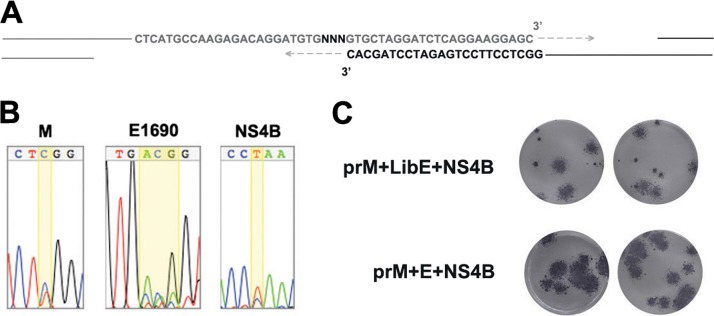
Construction of a DENV library with randomized amino acids at position E1690. (A) Diagram showing how the randomization was achieved at the assembly site of two PCR products (dark gray and black) during Gibson ligation. The primer sites for PCR are depicted in letters, while the rest of DNA strands are represented by solid lines. The arrows represent the direction of repair by Phusion polymerase during the assembly reaction. (B) Chromatograms of sequences at prM847, E1690, and NS4B7016 of prM+E+NS4B virus with amino acid randomization at E1690. The randomized position in E and heterogeneous nucleotides in M and NS4B are highlighted in yellow. (C) Comparison between the foci of the DENV library (prM+LibE+NS4B) and the foci of the virus with the same genetic background without randomization at E1690 (prM+E+NS4B). The foci for each virus stock in duplicate wells are shown.

With PCR products as the building blocks for viral infectious DNA, the approach can exploit various PCR techniques (error-prone PCR, DNA shuffling, and PCR with degenerate primers) to diversify virus sequences to produce mutant libraries. Screening and sorting of the libraries provide powerful methods to study the genetic basis of a phenotype. We created a virus library using a degenerate primer to randomize an amino acid at position E1690 (Fig. 4A). The mapping in Fig. 3 showed that the amino acid at E1690 has a strong influence on the focus size. Randomizing the amino acid at this position will generate a DENV library that can be used to study how the property of this critical amino acid affects focus size.

A DENV library was created by combining PCR products of prM, E (randomized at E1690), and NS4B from strain v17 and the rest from strain 4.1. Sequencing of the recovered viruses showed the scrambling of the sequence at the target position (Fig. 4B, E1690). The sequence chromatograms of prM847 (Fig. 4B, M) and NS4B7016 (Fig. 4B, NS4B) showed heterogeneity, as observed with the original DENV4-v17 stock (Fig. 1D). The focus assay of the DENV library indicated the presence of small-focus viruses that were not present when the amino acid at E1690 was not randomized in the same genetic background (Fig. 4C). Thus, by assembling the scrambled PCR products with those derived from heterogeneous viral cDNAs, this technique could directly diversify an existing pool of virus mutants. This capability is essential in directed-evolution experiments, where the mutant pool selected from each round is directly diversified for the next round of selection.

## DISCUSSION

The ease of assembling PCR products by Gibson assembly greatly simplifies genetic engineering of viral genomes. Traditional DNA ligation relies on complementary sticky ends generated by cutting DNA with restriction enzymes. The use of restriction enzymes has limited DNA assembly from multiple PCR products, as adding restriction sites must not interfere with the functions of the sequences at the joints. The restriction sites must also be unique to specifically ligate DNA. Seamless DNA assembly techniques, such as Gibson assembly (12), In-fusion (Agilent) (18, 19), SLIC (20), and SLiCE (21), could solve this problem by generating sticky ends on any linear DNA. Subsequent annealing of complementary single-stranded DNA overhangs and ligation of annealed fragments joins the DNA in a specific manner. Similar seamless assembly, such as CPEC (22) and SHA (23), relies on the annealing of cDNA strands at the ends to join DNA fragments during PCR amplification. In principle, any of these seamless DNA assembly techniques may be substituted for Gibson assembly in the virus construction approach presented here. Very recently, CPEC was applied to construct West Nile viruses in a similar bacterium-free approach for virus construction (24). The virus construction by CPEC could achieve similar recovery of the heterogeneity of a West Nile virus stock (24). Together, these results show the applicability of a seamless technique in virus recovery.

The ease of constructing viruses from multiple DNA fragments provided by this method can facilitate genetic mapping and screening of mutations (Fig. 3). The capability of Gibson assembly, as demonstrated in its application to the construction of mitochondrial genomes from oligonucleotides (25), would accommodate even finer fragmentation of viral genomes than ours (Fig. 1A) and make the approach applicable to RNA viruses with larger genomes, such as coronaviruses. While the data presented here show high accuracy of reconstructing viruses by this method, the possibility of unintended mutations caused by random PCR errors cannot be ruled out when viruses are constructed from PCR products. Confirming the identified mutations with additional methods and assays, such as full-genome sequencing of the derived viruses, will prevent such errors.

The virus construction approach described here addresses the throughput limitations of infectious clones. It shortens the construction time from months to days. It can recover the genetic diversity in the virus stock (Fig. 1B and D). It significantly reduces the amount of sequence verification required in a large-scale genetic analysis through the ease of recombining viruses (Fig. 3). It enables direct genetic manipulation of a virus mutant pool (Fig. 4). By eliminating these limitations, this approach supports large-scale forward genetics (Fig. 3) and expands our current ability to mutate viruses for reverse genetics and directed-evolution experiments (Fig. 4). Its capability is based on direct exploitation of PCR products amplified from viral cDNA as building blocks to construct infectious viral DNA in a single, highly efficient reaction of Gibson assembly. The throughput capability gained will support functional characterization of virus variants, which are being discovered at a breakneck pace by next-generation sequencing. Since it relies on a DNA-based expression format applicable to many positive-sense RNA viruses, its utility will be relevant to these viruses (13, 26, 27). The technique should accelerate vaccine and antiviral-drug development to fight against many pathogenic positive-sense RNA viruses.
